# Dissecting the Interplay Between NRF2 and BACH1 at CsMBEs

**DOI:** 10.3390/antiox14101203

**Published:** 2025-10-03

**Authors:** Maria-Armineh Tossounian, Alexander Zhyvoloup, Rakesh Chatterjee, Jerome Gouge

**Affiliations:** 1 Structural and Molecular Biology Department, University College London, London WC1E 6BT, UK; 2 Centre for Chemical Biology and Therapeutics, Institute for Stem Cell Science and Regenerative Medicine, Bellary Road, Bangalore 560065, India

**Keywords:** BACH1, NRF2, bZIP, redox regulation, protein-DNA interactions

## Abstract

BACH1 (BTB And CNC Homology 1) and NRF2 (Nuclear Factor Erythroid 2-related Factor 2) are transcription factors that regulate antioxidant and iron metabolism genes by competing for binding to cis-regulatory Maf-binding elements (CsMBEs) as heterodimers with small Maf proteins (sMafs). To dissect the mechanisms underlying this competition, we developed a chimeric tethering system where the DNA-binding domains of BACH1 or NRF2 were covalently linked to sMafG via a flexible, cleavable linker. This design enables efficient heterodimer formation on DNA and circumvents kinetic barriers to partner exchange in the solution. The site-specific fluorescent labelling of proteins allowed for the tracking of complex compositions by electrophoretic mobility shift assays. Both BACH1/sMafG and NRF2/sMafG heterodimers bind CsMBEs with similar affinities. Notably, DNA binding by BACH1 was impaired in a C574-dependent, redox-sensitive manner and promoted the exchange of heterodimer partners. Competition assays demonstrated that BACH1 and NRF2 can displace each other from preformed DNA-bound complexes, with greater efficiency when presented as preassembled heterodimers with sMafG. These findings reveal a redox-sensitive mechanism for regulating transcriptional switches at CsMBEs and highlight how preformed heterodimers facilitate the rapid displacement at target promoters.

## 1. Introduction

Cells must continuously adapt to fluctuating environmental and intracellular conditions, a process fundamentally governed by the regulation of gene expression at the transcriptional level [1]. Transcription factors and repressors orchestrate this adaptive response by binding to specific DNA sequences and modulating the recruitment of the transcriptional machinery, thereby activating or silencing gene expression as needed. This dynamic interplay is essential for maintaining cellular homeostasis, enabling responses to stress, and preventing pathological states. An important class of transcription factors involved in these processes is the basic leucine zipper (bZIP) family, characterised by a conserved basic region for DNA binding and a leucine zipper motif that mediates dimerization. The modularity of bZIP proteins allows them to form both homo- and heterodimers, greatly expanding their DNA-binding specificity and regulatory potential [2]. Among the bZIP superfamily, the Cap ‘n’ Collar (CNC) subfamily—including NRF2 (nuclear factor erythroid 2-related factor 2), BACH1 (broad complex, tramtrack, and bric-a-brac (BTB) and CNC homology 1), and small Maf proteins (sMafs, e.g., sMafF, sMafG, and sMafK)—plays a pivotal role in the transcriptional regulation of stress-responsive genes.

One of the most critical challenges for aerobic cells is the maintenance of redox balance. Reactive oxygen species, produced as byproducts of metabolism or in response to external insults, can cause significant cellular damage at elevated levels. To counteract oxidative stress, cells rely on a robust network of antioxidant defence genes, many of which are regulated by the NRF2 pathway [3]. Under basal conditions, NRF2 is sequestered in the cytoplasm by Keap1 (kelch-like enoyl-coenzyme A hydratase (ECH)-associated protein 1), which targets it for ubiquitin-mediated degradation. Upon oxidative or electrophilic stress, modifications of Keap1 cysteine residues disrupt the ubiquitination reaction [4]. This allows the newly synthesised NRF2 molecules to accumulate and translocate into the nucleus [5]. In the nucleus, NRF2 heterodimerises with sMaf proteins and binds to CNC-sMaf-binding element (CsMBE) [6] in the promoters of target genes. Among these, antioxidant response elements (AREs) represent a functionally important subset that drives the expression of genes involved in a broad cytoprotective programme.

Importantly, the CsMBEs are occupied by repressive complexes in the absence of stimulation. In fact, sMaf proteins act as repressors when forming homodimers because they lack transactivation domains. BACH1 can also form heterodimers with sMaf to bind CsMBEs in the promoter regions of iron metabolism genes (e.g., heme oxygenase 1 (HO-1), ferritin heavy chain 1, and ferritin light chain). These genes are uniquely regulated by hemes, which inhibit the DNA-binding ability of BACH1 [7,8] and promote its degradation [9,10]. This provides a rapid mechanism for gene derepression during cellular oxidative and heme stress. In keratinocytes, BACH1 knockdown was found to cause the robust derepression of HMOX1 and more modest increases in other NRF2 target genes (e.g., glutathione synthesis). This suggests that while KEAP1–NRF2 signalling is the dominant regulator of the antioxidant response, BACH1 could provide an additional layer of gene-specific repression [11]. Importantly, a possible second layer of redox regulation has been reported in NRF2 and BACH1. These proteins contain a conserved cysteine residue located near the DNA phosphate backbone that could act as a redox sensor. The C574S mutation in BACH1 abolishes its sensitivity to diamide, leading to persistent transcriptional repression even under oxidative conditions [12]. In contrast, the C506S mutation in mouse NRF2 (corresponding to Cys514 in humans) renders the transcription factor unable to activate gene expression in response to oxidative stress, likely due to the impaired DNA binding [13]. Together, these findings suggest that both transcriptional repressors and activators of CsMBE-driven genes are regulated by redox-sensitive mechanisms targeting their DNA-binding domains.

While NRF2 activation provides cytoprotection and suppresses carcinogenesis under physiological conditions, persistent NRF2 stabilisation—frequently due to mutations in Keap1 or NRF2—promotes tumour survival, metabolic adaptation, and chemoresistance [14]. BACH1, a transcriptional repressor, is recognised for its ability to drive tumour metastasis by repressing antioxidant genes and fostering pro-metastatic programmes [15]. Notably, recent work demonstrates that sustained NRF2 activation can stabilise BACH1 by inducing HO-1, thereby inhibiting BACH1 degradation and enhancing the metastatic potential, particularly in lung [16] and pancreatic cancers [17]. The elevated expression of both BACH1 and NRF2 correlates with a poor prognosis, emphasising the importance of their interplay at CsMBE promoters in cancer biology and therapy.

The structural basis for a DNA-bound sMafG homodimer [18] and DNA-bound NRF2/sMafG heterodimer [19] has been elucidated by crystallography. Both sMafG/sMafG and NRF2/sMafG utilise their leucine zippers to homo- and heterodimerise, whilst the basic regions of each monomer interact with the major groove of the CsMBE. These CNC-bZIP proteins share a similar overall architecture, with the CNC domain contributing to DNA-binding specificity and protein–protein interactions and the bZIP domain mediating dimerisation and DNA binding. Notably, bZIP proteins can form both homo- and heterodimers, and both sMafG and NRF2 have been shown to bind DNA as homodimers in vitro [19].

Previous studies have shown how NRF2 can outcompete the sMafG/sMafG homodimer to form an active NRF2/sMafG heterodimer, representing the activation of the promoters. However, to our knowledge, NRF2 outcompeting BACH1 has not been demonstrated in a reconstituted system mimicking the activation of iron metabolism genes. On the other hand, the re-inactivation of these genes (BACH1 replacing NRF2) has not been investigated. The roles of the oxidative modifications of cysteines in the DNA-binding domains of BACH1 remains to be fully elucidated with regard to the heme-sensing capabilities of BACH1. To address these gaps, we have adapted a tethering approach with a cleavable linker and fluorescent tagging to dissect the molecular mechanisms underlying the transition between repressed (BACH1/sMafG) and activated (NRF2/sMafG) states, as well as the re-inactivation process (NRF2/sMafG to BACH1/sMafG). Using competition assays, we observe that the displacement of the bZIP factors is facilitated when they compete as heterodimers instead of homodimers. In addition, the tethering system preserves the redox regulation of BACH1 through C574 oxidation, allowing us to probe how this post-translational modification influences BACH1’s DNA binding and promoter occupancy. We propose that the oxidation of BACH1 C574 acts as a secondary safety mechanism, preventing BACH1 from binding to CsMBE ARE promoters under oxidative conditions and ensuring the appropriate activation of antioxidant gene expression.

## 2. Materials and Methods

### 2.1. Cloning and Mutagenesis

Synthetic genes for full length BACH1, sMafG, and NRF2 have been codon-optimised and purchased from Genscript. The DNA-binding domains were subcloned in pET29 (BACH1residues 511–627, sMafG residues 1–162, and NRF2 residues 451–567) with a cleavable His-tag to yield the following constructs: BACH1^DBD^-3C-6xHis-pET29, 6xHis-TEV-sMafG^DBD^-pET29, and NRF2^DBD^-3C-6xHis-pET29.

Chimeric constructs were synthesised by fusing NRF2^DBD^ or BACH1^DBD^ with sMafG^DBD^ gene to obtain NRF2-sMafGχ (NRF2^DBD^-3C-6xHis-TEV-sMafG^DBD^χ-pET29) and BACH1-sMafGχ (BACH1^DBD^-3C-6xHis-TEV-sMafG^DBD^χ-pET29), respectively. The sequence of the linker is as follows: GSGLEVLFQGPGSAGSAAGSGEGSAGHHHHHHGSAGSAAGSGHHHHHHGSASAAGSGESAGASEGSGAASGASGEGSAGSENLYFQGG. Point mutation of BACH1^C574A^-sMafGχ was generated by divergent PCR.

YbbR containing chimeric constructs were generated by introducing a YbbR tag (DSLEFIASKLA) to the C-terminus of the NRF2-sMafGχ and Bach1-sMafGχ constructs using divergent PCR. The generated constructs are NRF2-sMafGχ^YbbR^ (NRF2^DBD^-3C-6xHis-TEV-sMafG^DBD^χ-YbbR-pET29) and BACH1-sMafGχ^YbbR^ (BACH1^DBD^-3C-6xHis-TEV-sMafG^DBD^χ-YbbR-pET29). To observe different gel shifts during competition assays, we generated NRF2^DBD^-6xHis-TEV-sMafG^DBD^χ-YbbR-pET29 and BACH1^DBD^-6xHis-TEV-sMafG^DBD^χ-YbbR-pET29 constructs by removing the 3C protease cleavage site using divergent PCR.

### 2.2. Protein Expression and Purification of the DNA-Binding Domain and Chimeric Constructs

The DBD constructs were expressed at 30 °C for 3 h 30 min in *E. coli* One Shot™ BL21 Star™ cells (DE3; Invitrogen, Waltham, MA, USA) using 1 mM IPTG when the optical density (OD_600_) reached 0.7. The chimeric constructs were expressed overnight (O/N) at 20 °C with 1 mM IPTG supplemented with appropriate antibiotics. After expression, the cells were harvested by centrifugation using a Beckman Coulter JLA8.1000 rotor (5000 rpm; Amersham, UK) for 15 min at 4 °C. The pellet was resuspended in the resuspension buffer (50 mM HEPES pH 7.9, 500 mM NaCl, 10% glycerol, 2 mM ß-mercaptoethanol, 10 mM MgCl_2_, 40 mU/mL DNaseI-XT (NEB), and 5 mM imidazole and protease inhibitor cocktail (Roche, Mannheim, Germany)). After resuspension, cells were sonicated using Soniprep 150 (5 s pulse ON/5s pulse OFF; 10 min at 35% amplitude). Following sonication, the sample was centrifuged using the Beckman CoulterJA-25.50 rotor (18,000 rpm) for 30 min at 4 °C.

Protein purification was performed using the ÄKTA Pure™ system (Cytiva, Amersham, UK). The filtered lysate was loaded onto a HisTrap™ HP column (Cytiva) equilibrated in binding buffer (50 mM HEPES 7.9, 500 mM NaCl, 10% glycerol, 2 mM ß-mercaptoethanol, and 10 mM imidazole). The column was washed with 50 mM HEPES 7.9, 500 mM NaCl, 10% glycerol, 2 mM ß-mercaptoethanol, and 50 mM imidazole. Proteins were eluted with 50 mM HEPES pH 7.9, 250 mM NaCl, 10% glycerol, 2 mM ß-mercaptoethanol, and 300 mM imidazole. The eluted protein was then diluted to have a lower NaCl concentration and loaded onto a HiTrap™ Heparin HP (Cytiva) column, equilibrated in 50 mM HEPES pH 7.9, 10% glycerol, 2 mM DTT, and 250 mM NaCl. The protein was eluted using a step elution with 1 M NaCl in the same buffer. The eluted protein was further purified using size exclusion chromatography. The protein was injected in Superdex™ 200 Increase 16/600 column (Cytiva), equilibrated in 50 mM HEPES pH 7.9, 10% glycerol, 2 mM TCEP, and 500 mM NaCl. The purity of the sample was assessed using SDS-PAGE gel. The protein concentration was determined using the Pierce™ Bradford plus protein assay reagent (Thermo Fisher, Waltham, MA, USA), and the protein was flash frozen and stored at −80 °C. All the proteins were expressed and purified similarly, except for NRF2^DBD^, which was purified by His-tag purification followed by Heparin purification.

### 2.3. Protein Expression and Purification of TEV, 3C, and Sfp

Tobacco Etch Virus (TEV) protease, HRV 3C (3C) protease, and 4′-phosphopantetheinyl transferase (Sfp) were expressed overnight at 20 °C with 1 mM IPTG in *E. coli* One Shot™ BL21 Star™ cells (DE3; Invitrogen). These constructs have been gifted by Alessandro Vannini. After expression, cells were harvested and sonicated as described above, excluding the use of protease inhibitors within the lysis buffers. Protein purification was performed using the ÄKTA Pure™ system (Cytiva).

The 3C lysate was loaded onto a HisTrap™ HP column (Cytiva) equilibrated in binding buffer (25 mM HEPES 7.9, 500 mM NaCl, 10% glycerol, 1 mM ß-mercaptoethanol, and 10 mM imidazole). The column was washed with 15% elution buffer, followed by a step elution with 100% elution buffer (25 mM HEPES pH 7.9, 250 mM NaCl, 10% glycerol, 1 mM ß-mercaptoethanol, and 300 mM imidazole). The eluted protein was diluted to lower the NaCl concertation. The protein was then loaded onto HiTrap™ SP HP column (Cytiva) equilibrated in 8% elution buffer (25 mM HEPES pH 7.9, 160 mM NaCl, 10% glycerol, and 1 mM DTT), followed by a gradient elution to 50% elution buffer (25 mM HEPES pH 7.9, 1 M NaCl, 10% glycerol, and 1 mM DTT). The purity of the purified sample was assessed using SDS-PAGE gel. The protein concentration was determined using the Bradford assay reagent and the protein was flash frozen and stored at −80 °C. The TEV lysate was passed onto a HisTrap™ HP column (Cytiva) equilibrated in binding buffer (25 mM HEPES 7.9, 500 mM NaCl, 10% glycerol, 1 mM ß-mercaptoethanol, and 10 mM imidazole). The column was washed with 15% elution buffer, followed by a step elution with 100% elution buffer (25 mM HEPES pH 7.9, 250 mM NaCl, 10% glycerol, 1 mM ß-mercaptoethanol, and 300 mM imidazole). Sfp (phosphopantetheinyl transferase) was purified as described previously [18].

### 2.4. SEC-MALS Analysis of Protein Oligomeric State

To assess the oligomeric state of BACH1^DBD^ and sMafG^DBD^, we performed size exclusion chromatography coupled with multiangle light scattering (SEC-MALS; Agilent 1100, Wyatt Dawn8+, T-rEX; Cheadle, UK). The Superdex™ 75 increase 10/300 column (Cytiva) was equilibrated in 50 mM HEPES pH 7.9, 2 mM TCEP, and 500 mM NaCl. The protein (1–2 mg) was injected into the system, and the chromatograms obtained were exported and analysed by GraphPad Prism (version 10).

### 2.5. CoA–Fluorescein Conjugation and Protein Labelling

The CoA–fluorescein conjugate was generated as described before [20]. Briefly, fluorescein-5-maleimide (; 1.2 mg in 500 μL DMSO, Thermo Fisher) and coenzyme A (CoA) trilithium salt (BIC7401; Apollo Scientific, Manchester, UK; and 3.2 mg in 1500 μL 100 mM sodium phosphate pH 7.0 mM) were incubated at 25 °C for 1 h in the dark. HPLC (Waters, Elstree, UK) was used to purify the CoA–fluorescein conjugate. The sample was centrifuged to remove precipitants and injected onto a 10 × 250 mm C8 Vydac column (Cat. 208TP1010) equilibrated by 2.5% acetonitrile in 200 mM ammonium acetate pH 6. The CoA–fluorescein conjugate was eluted using isocratic elution (200 mM ammonium acetate pH 6 and 2.5% acetonitrile) from time 0–2 min, followed by gradient elution (2.5–32.5% acetonitrile in 200 mM ammonium acetate pH 6) from time 2–17 min at 6 mL/min flow rate. The CoA–fluorescein conjugate peak was detected at 215 nm and 260 nm. The eluted peak (at 11.2 min) was then lyophilized and stored at −20 °C. Prior to protein labelling, the lyophilized CoA–fluorescein conjugate was resuspended in water, and its concentration was measured using the Nanodrop 2000c (Thermo Fisher).

To label the YbbR tag containing constructs (NRF2-sMafGχ^YbbR^, BACH1-sMafGχ^YbbR^, NRF2-TEV-sMafGχ^YbbR^, and BACH1-TEV-sMafGχ^YbbR^) with fluorescein, a 1:2:0.2 ratio of protein (1 mg)/CoA–fluorescein conjugate/Sfp was used. The mixture was incubated for 2 h at 25 °C in the dark. To separate the labelled protein Sfp, the sample was diluted with a low NaCl-containing buffer and then applied onto a HiTrap™ Heparin column (1 mL, Cytiva), equilibrated in 50 mM HEPES pH 7.9, 10% glycerol, 1 mM TCEP, and 250 mM NaCl. The protein was eluted using a gradient elution of 0–75% elution buffer (100% elution buffer: 50 mM HEPES pH 7.9, 10% glycerol, 1 mM TCEP, and 2 M NaCl) for 20 min. Both 280 nm and 488 nm were monitored. The purity of the sample was assessed using SDS-PAGE gel. The protein concentration was measured by Bradford assay and nanodrop. The labelled proteins were stored at −80 °C.

### 2.6. Electrophoretic Mobility Shift Assays (EMSAs)

EMSAs were used to study the protein–DNA interactions. A 30-mer sequence of fluorescently labelled Forward (5′-[Fluo]-ctcgggaccgTGACTCAGCAggaaaaacac-3′) and a non-labelled Reverse oligonucleotide (5′gtgtttttccTGCTGAGTCAcggtcccgag-3′) were synthesised by Sigma (Merck Life Science, Gillingham, UK). This sequence, including the flanking regions, is based on naturally occurring CsMBE found in the HMOX-1 promoter (GRCh37/hg19 chr22:35,768,001–35,768,031 or GRCh38/hg38 chr22:35,372,008–35,372,037) and known to be bound by BACH1 [21] and NRF2 [22] in ChIP-seq. The fluorophore on the Fw oligo is either fluorescein or Cy5, as indicated for each experiment. The oligos were annealed using the Biorad thermal cycler (Watford, UK).

To determine the affinity constants (K_d_) of the chimeric constructs (NRF2-sMafGχ and BACH1-sMafGχ), increasing concentrations of the proteins (0–300 nM) were mixed with the fluorescein labelled dsDNA (3 nM) in the reaction buffer (150 mM NaCl, 50 mM HEPES pH 7.9, 10% glycerol, 1 mM TCEP, 1 mM DTT, and 2 mM MgCl_2_). The samples were incubated with TEV (50 μg/mL) and 3C (50 μg/mL) proteases for 4 h at 25 °C and then loaded on a 6% non-denaturing polyacrylamide gel. A similar experimental setup was used to determine the relative K_d_ of NRF2^DBD^, BACH1^DBD^, and sMafG^DBD^ (0–3 μM). Independent triplicates were performed for each experiment.

To compare the migration of the different chimeric constructs (NRF2-sMafGχ, NRF2-TEV-sMafG^YbbR^, BACH1-sMafGχ, and BACH1-TEV-sMafG^YbbR^), the proteins were incubated with Cy5-labelled dsDNA (3 nM) and TEV (50 μg/mL) and 3C (50 μg/mL) proteases for 4 h at 25 °C. As a control, the DBD-containing proteins (NRF2^DBD^, BACH1^DBD^, and sMafG^DBD^) were also used. The samples were then loaded on a 10% non-denaturing polyacrylamide gel. Independent duplicates were performed.

### 2.7. Time-Course Experiment on C574 Redox Sensitivity

Prior to the time-course protein oxidation experiment, the reducing agent was removed from the sample by gel filtration (Superdex™ 200 increase 5/150 column, Cytiva) using a buffer containing 50 mM HEPES pH 7.9, 10% glycerol, and 500 mM NaCl. The eluted sample was flash frozen and stored at −80 °C.

Two experimental setups were used. In the “incubation-first experiment”, BACH1-sMafGχ or BACH1^C574A^-sMafGχ (200 nM) was incubated under non-reducing conditions (150 mM NaCl, 50 mM HEPES pH 7.9, 10% glycerol, and 2 mM MgCl_2_) at 4 °C for 0, 4, 8, or 10 h. Proteins were then mixed with fluorescein-labelled dsDNA (3 nM) and TEV/3C proteases (50 μg/mL each) and incubated for 4 h at 25 °C. A parallel 10 h sample was supplemented with 5 mM DTT.

In the “assembly-first experiment”, BACH1-sMafGχ (200 nM) was first assembled with fluorescein-labelled dsDNA (3 nM) and TEV/3C proteases (50 μg/mL each), followed by incubation at 4 °C for 0, 4, 8, or 10 h. A 10 h sample with 5 mM DTT was included as control. All samples were analysed on 6% non-denaturing polyacrylamide gels. Incubation-first assays were performed in ≥3 independent replicates, and assembly first assays in two replicates.

### 2.8. Competition Assays Using EMSA

To perform competition assays, fluorescein-labelled NRF2-TEV-sMafGχ^YbbR^ (150 nM) was incubated with Cy5-labelled dsDNA (3 nM) and TEV (50 μg/mL) and 3C (50 μg/mL) proteases for 4 h at 25 °C. Following the incubation, increasing concentrations of BACH1^DBD^ (150 nM-1.5 μM) or BACH1-sMafGχ (25–300 nM) were added, and the samples were incubated O/N at 4 °C. Similarly, fluorescein-labelled BACH1-TEV-sMafGχ^YbbR^ (150 nM) was incubated with Cy5-labelled dsDNA and TEV/3C proteases for 4 h at 25 °C. Following the incubation, increasing concentrations of NRF2^DBD^ (150 nM–1.5 μM) or NRF2-sMafGχ (25–300 nM) were added, and the samples were incubated O/N at 4 °C. For the titration by DBD proteins, either NRF2-sMafGχ^YbbR^ or BACH1-sMafGχ were used as controls. For the titration by chimeric constructs, NRF2-sMafGχ or BACH1-sMafGχ were used as controls in the absence of the fluorescein-labelled proteins. All the samples were then loaded on a 10% non-denaturing polyacrylamide gel. Independent duplicates were performed for each experiment.

### 2.9. BACH1/sMafG Displacement by NRF2/sMafG Under Non-Reducing Conditions

Bach1/sMafG was buffer exchanged as described above. In the “assembly-first experiment”, BACH1-sMafGχ (200 nM) was preassembled with Cy5-labelled dsDNA (3 nM; in presence of TEV/3C) and then incubated at 4 °C for 8 h under non-reducing conditions. In the “incubation-first experiment”, BACH1-sMafGχ (200 nM) was incubated at 4 °C for 8 h under non-reducing conditions and then assembled with Cy5-labelled dsDNA (3 nM; in presence of TEV/3C) for 4 h at RT. For both experiments, the BACH1/sMafG/dsDNA complex was incubated O/N with increasing concentrations of fluorescein-labelled NRF2-TEV-sMafG^YbbR^ at 4 °C. All the samples were then loaded on a 10% non-denaturing polyacrylamide gel. At least two independent replicates were performed for each experiment.

### 2.10. EMSA Running, Scanning, and Processing

The EMSAs with 6% and 10% non-denaturing polyacrylamide gels were ran in 1X TBE buffer at 4 °C, 40 mA for 17 min or 30 min, respectively. The gels were imaged by the Amersham Typhoon 5 (Cytiva) using the Cy5 670BP30 and Cy2 525BP20 filters, which detect the Cy5 and fluorescein signals, respectively. Band intensity quantification was performed by ImageQuant™ analysis software (version 11.0; Cytiva). To determine the K_d_ values, the protein concentration in function of %Protein bound to DNA was plotted using GraphPad Prism10. The curve was fitted to a non-linear regression, and the K_d_ value was obtained. Bands marked with an asterisk correspond to degradation products (figure in the Section 3.5), incomplete linker cleavage (figure in Section 3.4), non-specific species, or combination of these (Figure S3).

### 2.11. BACH1/sMafG/CsMBE Structure Prediction

Protein structures were predicted using AlphaFold 3 server [23]. Sequences of the DNA-binding domains (BACH1 residues 511–627, sMafG residues 1–162) as well as CsMBE-containing sequences (5′-ctcgggaccgTGACTCAGCAggaaaaacac-3′ and 5′-gtgtttttccTGCTGAGTCAcggtcccgag-3′) were used as inputs. Predicted structures were assessed using pLDDT (most of the structure reached >90, especially around C574), ipTM (0.78, suggesting that the predicted structure is close to experimental ones), and PAE (shows very low expected error around C574).

### 2.12. Sequence Alignments

Sequences were retrieved from Uniprotkb with the following identifiers: Q16236 (NF2L2_HUMAN, human NRF2), O15525 (MAFG_HUMAN, human sMafG), 14867 (BACH1_HUMAN, human BACH1), P97302 (BACH1_MOUSE, mouse BACH1), A0A4X1U9G6 (A0A4X1U9G6_PIG, pig BACH1), F7BYY6 (F7BYY6_XENTR, xenopus BACH1), and A0AB32T1F9 (A0AB32T1F9_DANRE, zebrafish BACH1). Sequences were aligned using Clustal omega (version 1.2.4) [24]; the alignments were prepared using ESPript (version 3.0) [25].

### 2.13. Crosslinking Using Hanging-Drop Vapour Diffusion Method

To determine the oligomerization states of BACH1^DBD^ and sMafG^DBD^, the proteins were crosslinked by glutaraldehyde using the hanging-drop vapour diffusion method [26]. Briefly, 120 μL at 5% for BACH1^DBD^ or 25% for sMafG^DBD^ was added into the wells of a 48-well crystallisation plate, and 5–7 μM of protein (10 μL) was added onto a cover slip. Wells were covered by the cover slip, and the sample was incubated for 15 min (sMafG^DBD^) or 45 min (BACH1^DBD^) to allow for vapour diffusion and protein crosslinking. Following incubation, 250 mM ammonium sulfate was added to stop the crosslinking reaction. The sample was then mixed with SDS-loading dye, boiled, and analysed on SDS-PAGE using Coomassie staining (Instant Blue; Expedeon Ltd., Swavesey, UK).

## 3. Results

### 3.1. Recombinantly Expressed BACH1^DBD^ and sMafG^DBD^ Are Homodimers in Solution and Can Bind CsMBE

**Figure 1:**
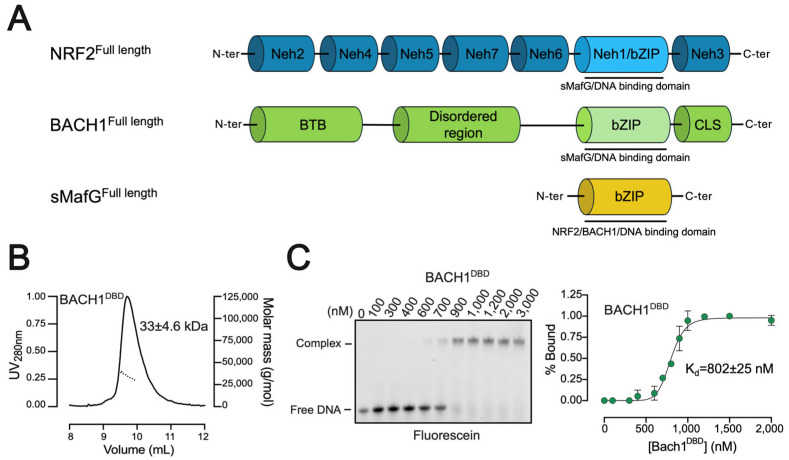
Domain organisation of NRF2, BACH1, and sMafG and BACH1^DBD^ interaction with CsMBE. (**A**) Representation of the different domains of the full length NRF2, BACH1, and sMafG. (**B**) SEC-MALS chromatogram of BACH1^DBD^ (33 ± 4.6 kDa) shows the homodimeric state of the recombinant protein. (**C**) Fluorescence-based EMSA gel of BACH1^DBD^ using fluorescein-dsDNA. The protein–DNA complex and the free DNA bands are indicated. The concentration values represent the concentration of the homodimeric form of the protein. The band intensity analysis is shown on the right. The apparent affinity of BACH1^DBD^ binding to CsMBE is estimated to be around 802 ± 25 nM. Data presented are from three independent replicates.

The leucine zippers of bZIP transcription factors are the primary mediators of protein–protein interactions both in a solution and on DNA [27]. Residues flanking either side of the coiled coil can also contribute to the dimerisation process [28]. It is plausible that recombinant bZIP domains form homodimers upon expression, whether they are functional (e.g., sMafG) or not (e.g., NRF2 and BACH1). To assess the homodimerisation potential of NRF2, BACH1, and sMafG, we expressed and purified their respective DNA-binding domains (NRF2^DBD^, BACH1^DBD^, and sMafG ^DBD^) (Figure 1A and Figure S1A). We first subjected each DBD to glutaraldehyde vapour diffusion crosslinking [29]. Using this method, we observed that both BACH1^DBD^ and sMafG^DBD^ readily formed homodimers, as indicated by the increased apparent molecular weight on the SDS–PAGE (Figure S2A). In contrast, no homodimer formation was detected for NRF2^DBD^ despite extensive screening crosslinking attempts. Because chemical crosslinking can stabilise transient interactions, we validated these findings by SEC–MALS (Figure 1B and Figure S2B). The measured molecular masses—33 ± 4.6 kDa for BACH1^DBD^ (Figure 1B) and 34 ± 0.8 kDa for sMafG^DBD^—were consistent with homodimeric assemblies.

In the absence of oxidative stress, cytoprotective genes controlled by NRF2 are known to be repressed by homodimeric sMafG, suggesting that sMafG homodimers are physiologically relevant. A previous study also demonstrated that NRF2^DBD^ can bind to the DNA as a homodimer, albeit at a lower affinity than sMafG^DBD^ [19]. Importantly, NRF2 binding the DNA as a homodimer is deemed to be artefactual. To assess whether BACH1^DBD^ exhibits a similar capacity, we performed a fluorescence-based EMSA. The titration of BACH1^DBD^ on DNA led to a clear DNA shift, indicating the formation of a complex (Figure 1C). Quantification of the bound fraction across protein concentrations allowed us to estimate the apparent DNA-binding affinity (K_d_) of BACH1^DBD^ (802 ± 25 nM, Figure 1C). We also confirmed that, in our hands, both sMafG^DBD^ and NRF2^DBD^ bind to the DNA as homodimers (Figure S3). Their corresponding apparent affinities were 203 ± 6 nM for sMafG^DBD^ and 590 ± 79 nM for NRF2^DBD^. Together, these data show that all three bZIP domains are capable of binding to the CsMBE sequence, although with markedly different affinities. We propose that the apparent binding of homodimeric BACH1^DBD^ to CsMBE sites is an artefact, analogous to the homodimeric binding sometimes observed with NRF2^DBD^. Indeed, it is admitted that BACH1 requires a partner to bind the DNA [30] and, more specifically, sMaf proteins to recognise CsMBE sites in vivo [8].

### 3.2. Tethered NRF2/sMafG and BACH1/sMafG Reveal Heterodimer Assembly and Higher DNA-Binding Affinity

Considering that sMafG^DBD^, NRF2^DBD^, and BACH1^DBD^ can each bind to the DNA as homodimers, determining the binding affinity of the heterodimers (NRF2/sMafG and BACH1/sMafG—both bona fide CsMBE binders [30]) is not straightforward. Accurate estimation would require expressing each bZIP domain separately, mixing them at equimolar ratios, and relying on the dissociation and reassociation of the leucine zipper interfaces to enable heterodimer formation on the DNA. This approach is complicated by the need for the spontaneous reorganisation of dimers, which may not occur efficiently under experimental conditions.

**Figure 2:**
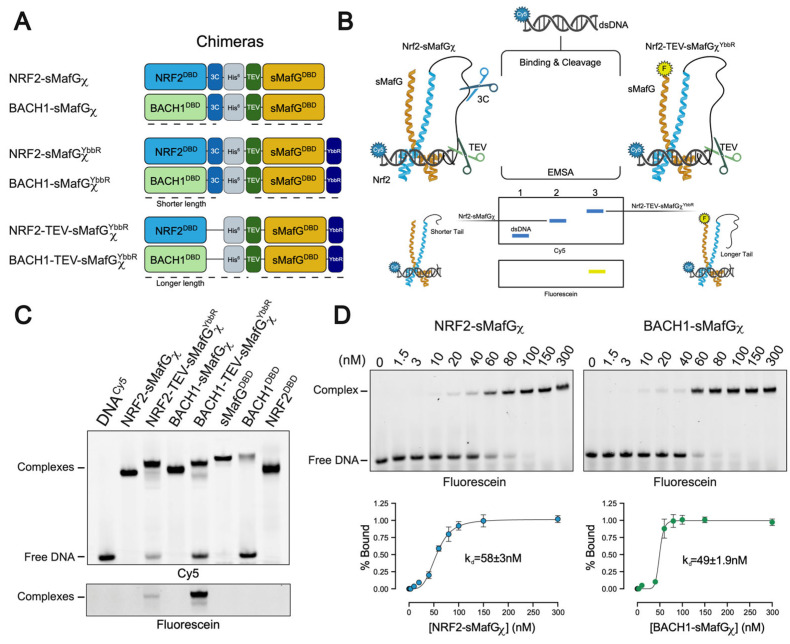
NRF2^DBD^, BACH1^DBD^, and sMafG^DBD^ chimeric construct designs and affinity determinations. (**A**) Design of chimeric constructs. The YbbR tag added at the C-terminus of sMafG allows for fluorescent labelling. The dotted lines represent constructs following protease cleavage. (**B**) Schematic representation of the binding of the NRF2-sMafGχ and NRF2-TEV-sMafGχ^YbbR^ to the Cy5-labelled DNA in the presence of 3C and TEV proteases, which cut the linker between NRF2^DBD^ and sMafG^DBD^. The TEV (green scissor) and 3C (blue scissor) cleavage sites and fluorescence tag (yellow circle; fluorescein and blue circle; and Cy5) are indicated. The difference in cleavage sites causes different migrating bands (Cy5 signal) on EMSA, which demonstrates the presence of Nrf2^DBD^, while the presence of a band with fluorescein signal demonstrates the presence of sMafG^YbbR^. The image was generated using BioRender. (**C**) EMSA comparing the mobility of the chimeric constructs (NRF2-sMafGχ^YbbR^, BACH1-sMafGχ^YbbR^, and fluorescein-labelled NRF2-TEV-sMafGχ^YbbR^ and BACH1-TEV-sMafGχ^YbbR^). The upper panel represents the Cy5-labelled DNA, and the lower panel corresponds to fluorescein signal on sMafG^DBD-YbbR^. The different migration and presence of fluorescein signals confirm the heterodimeric assembly on the DNA. The difference in fluorescein absorption in the lower panel is explained by different labelling efficiency. Data presented are from two independent replicates. (**D**) EMSA showing the binding of NRF2-sMafGχ and BACH1-sMafGχ on fluorescein-labelled DNA. The apparent affinity constants of NRF2-sMafGχ (k_d_ = 58 ± 3 nM) and BACH1-sMafGχ (k_d_ = 49 ± 1.9 nM) are shown. Data presented are from three independent replicates.

To circumvent this issue, we employed a tethering strategy previously described [30] with some modifications. Chimeric constructs (χ) were generated by genetically fusing NRF2^DBD^ (or BACH1^DBD^) to sMafG^DBD^ via a flexible linker containing a 6×His tag, which is flanked by two protease cleavage sites (Figure 2A). These yielded NRF2-sMafGχ and BACH1-sMafGχ chimeric constructs, respectively. To confirm that the chimeric proteins can reconstitute heterodimeric DNA binding following the linker cleavage, we generated two constructs lacking the 3C protease site. This ensured the accurate tracking of NRF2 (and BACH1) by generating a supershift (Figure 2B,C). Indeed, the doubly cleaved heterodimer is expected to migrate faster than the singly cleaved version (Figure 2B). Additionally, we inserted a YbbR tag that allows for the fluorescent labelling of sMafG by Sfp [20] (see Section 2). Fluorescein-specific scanning of native gels thus enables the direct and specific tracking of sMafG within protein–DNA complexes (Figure 2B). This yielded the constructs NRF2-TEV-sMafGχ^YbbR^ and BACH1-TEV-sMafGχ^YbbR^.

Using this strategy, chimeric proteins (NRF2-sMafGχ and BACH1-sMafGχ), along with their corresponding fluorescein-labelled controls, were incubated with Cy5-labelled DNA and the relevant proteases (Figure 2C). In EMSA, the comparison of the NRF2-sMafGχ migration (lane 2) with NRF2-TEV-sMafGχ^YbbR^ (lane 3) revealed a shift that can be attributed to the linker being double cleaved. The fluorescein signal in lane 3 confirmed the presence of sMafG^YbbR^ in the complex. These observations demonstrate that both NRF2^DBD^ and sMafG^DBD^ are present and participate in DNA binding as a heterodimer. A similar behaviour was observed for BACH1-sMafGχ (lane 4) versus BACH1-TEV-sMafG^YbbR^ (lane 5), confirming the BACH1/sMafG heterodimerisation on DNA (Figure 2C).

Because the tethered constructs eliminate the need for the dissociation and reassembly of bZIP domains, they enable the direct measurement of the heterodimer-binding affinity. Titration of NRF2-sMafGχ and BACH1-sMafGχ onto fluorescein-labelled CsMBE-containing dsDNA produced a single, slower migrating complex across the concentration range, suggesting specific heterodimer formation (Figure 2D). Quantification of the bound DNA over increasing protein concentrations yielded an apparent K_d_ of 58 ± 3 nM for NRF2-sMafGχ and 49 ± 1.9 nM for BACH1-sMafGχ (Figure 2D). These affinities are higher than those observed for the homodimeric DBDs (Figure 1C and Figure S3), reinforcing the idea that the heterodimers are bona fide DNA binders.

### 3.3. Redox Sensitivity of BACH1^DBD^ C574 Modulates DNA Binding

**Figure 3:**
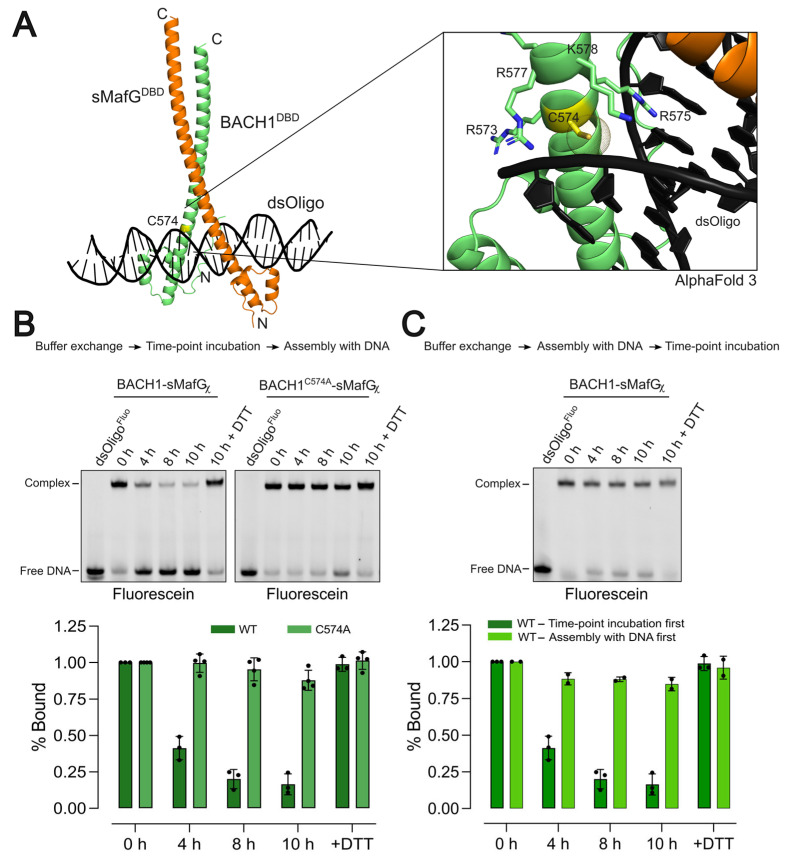
Redox regulation of BACH1^DBD^ by C574. (**A**) AlphaFold 3 model of the heterodimeric BACH1^DBD^/sMafG^DBD^ (green/orange) in complex with CsMBE-containing dsDNA (black). BACH1^DBD^ C574 (yellow sticks; dotted sphere) makes van der Walls contacts with the DNA and is surrounded by positively charged residues of the basic region (R573, R575, R577, and K578; Figure S1B). (**B**) Incubation-first experiment. BACH1-sMafGχ (WT) and BACH1^C574A^-sMafGχ (C574A) were incubated at 4 °C in absence of reducing agents for increasing period, then assembled with dsDNA. EMSA gel analysis of BACH1-sMafGχ shows a time-dependent decrease in complex formation that addition of DTT recovers. In the same conditions, the C574A mutant is insensitive to oxidation. The histogram shows the band intensity of time-dependent binding of heterodimer–DNA complex. Data presented are from at least three independent replicates. (**C**) Assembly first experiment. BACH1/sMafGχ/DNA complex was assembled in absence of reducing agents, then incubated for increasing period. EMSA analysis of BACH1-sMafGχ shows that the complex is virtually insensitive. The histogram shows the band intensity of time-dependent binding of heterodimer–DNA complex. Data presented are from at least two independent replicates.

A conserved cysteine residue, C574 (Figure S1B), within the DNA-binding domain of BACH1 is shown to be reactive towards electrophilic and oxidative compounds such as 4-hydroxynonenal (HNE) [12], diamide [12], and arsenite [31] in cells. To explore the structural basis of this redox sensitivity, we used AlphaFold 3 to model the BACH1^DBD^/sMafG^DBD^/CsMBE complex. The predicted structure revealed that C574 is positioned near the DNA backbone and is flanked by several positively charged residues (Figure 3A). This configuration resembles that of other redox-sensitive transcription factors, where redox-sensitive cysteines interact closely—through van der Waals contacts—with the DNA (e.g., NF-kB [32] and Brf2 [33]; Figure S4).

We tested whether exposure to molecular oxygen under non-reducing conditions could reveal the redox sensitivity of C574 and alter the DNA-binding activity. This was assessed using a time-course oxidation assay with a BACH1-sMafGχ construct. The fusion protein was incubated at 4 °C in the absence of reducing agents (see Section 2) for increasing periods of time, then DNA was added, and finally, the complex formation was assessed by EMSA. This experiment is referred to as “incubation-first”. We observed a time-dependent decrease in the intensity of the protein–DNA complex. After 10 h in the absence of reducing agents, the BACH1^DBD^/sMafG^DBD^ complex could no longer bind to DNA. Importantly, DNA binding was restored by the addition of 5 mM DTT (Figure 3B). In similar conditions, the BACH1^C574A^-sMafGχ mutant was unaffected by the absence of reducing agents, indicating that the redox sensitivity is specifically mediated by C574 (Figure 3B). These results suggest that the oxidation of C574 directly impairs BACH1’s ability to bind DNA.

To further examine the redox sensitivity of C574, we removed the reducing agent from purified BACH1-sMafGχ and first assembled the heterodimer on DNA, then incubated it under non-reducing conditions for increasing periods of time before analysing the species on EMSAs. This experiment is referred to as “assembly-first”. In contrast to the “incubation-first” experiment (Figure 3B), the intensity of the DNA–protein complex band stayed virtually the same over time, suggesting that the binding remained stable (Figure 3C). This suggests that the oxidative modifications at C574, which occur when BACH1/sMafG is off the DNA, may not take place when the complex is bound to DNA or that the modifications are not sufficient to release BACH1 binding.

### 3.4. Competition Assays with DNA-Binding Domains

We sought to investigate the mechanism of bZIP replacement at the promoter. Indeed, under oxidative conditions, NRF2^DBD^ can displace a preformed BACH1/sMafG/DNA complex. Conversely, during recovery from stress, the repression of CsMBE-regulated genes involved in iron metabolism would require BACH1^DBD^ to replace NRF2^DBD^ from the DNA.

**Figure 4:**
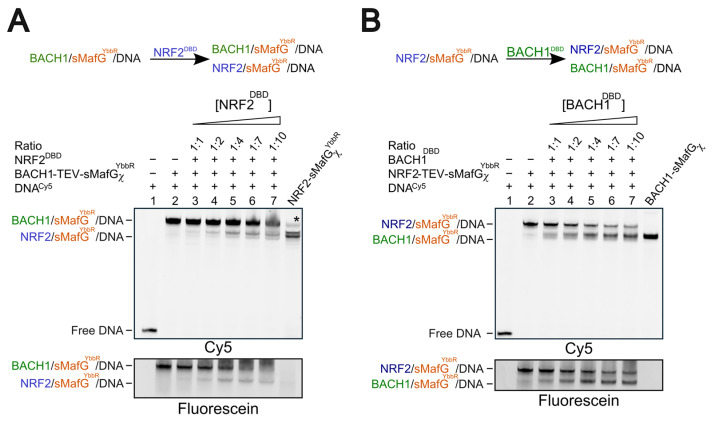
Competitive displacement by the DNA-binding domains. (**A**) Titration of NRF2^DBD^ over BACH1/sMafG^YbbR^/DNA. NRF2^DBD^ outcompetes BACH1^DBD^ to bind sMafG^YbbR^/DNA, as demonstrated by a faster migrating complex that is labelled with fluorescein (sMafG^YbbR^). (**B**) Titration of BACH1^DBD^ over NRF2/sMafGχ^YbbR^/DNA. BACH1^DBD^ outcompetes NRF2^DBD^ to bind sMafG^YbbR^/DNA, as demonstrated by a faster migrating complex that is labelled with fluorescein (sMafG^YbbR^). The ratio indicates the complex bound on the DNA (BACH1/sMafG^YbbR^ for A or NRF2/sMafG^YbbR^ for B) over the titrated DBD (NRF2^DBD^ in (**A**) and BACH1^DBD^ in (**B**)). As a control, DNA-bound NRF2/sMafG^YbbR^ (**A**) and BACH1/sMafG (**B**) were used. The black asterisk represents non-specific bands. NRF2-sMafG^YbbR^ has a low derivatisation efficiency, explaining the low intensity of the fluorescein band in NRF2-sMafGχ^YbbR^ in panel (**A**).

To recapitulate the promoter activation, we first pre-incubated fluorescein-labelled BACH1-TEV-sMafGχ^YbbR^ (Figure 2A) with CsMBE-containing Cy5-labelled DNA and titrated increasing amounts of NRF2^DBD^ (Figure 4A). The successful displacement of BACH1^DBD^ would manifest as follows: (i) a faster migrating complex due to the lower molecular weight of NRF2^DBD^ (Figure 2A), and (ii) a downward shift in the fluorescein-labelled sMafG^YbbR^ signal, indicating the formation of the heterodimer NRF2/sMafG^YbbR^. Upon titration with NRF2^DBD^, a distinct faster migrating complex was observed at a 1:2 ratio (1 BACH1/sMafG^YbbR^ for 2 NRF2^DBD^), along with a shift in the fluorescein signal position. This displacement appeared to plateau even at a 10-fold excess of NRF2^DBD^ over BACH1/sMafG^YbbR^, suggesting that, in these conditions, NRF2^DBD^ can only partially displace BACH1^DBD^ from the sMafG^YbbR^/DNA complex.

We then performed the reverse experiment by titrating BACH1^DBD^ (Figure 4B) into a preformed fluorescein-labelled NRF2/sMafG^YbbR^/DNA complex (Figure 2A). This scenario recapitulates the inactivation of the promoter. Similarly to the previous experiment, we observed a faster migrating complex and a downward shift in the fluorescein signal, indicating that BACH1^DBD^ can displace NRF2^DBD^ to bind sMafG^YbbR^/DNA. Interestingly, NRF2 was displaced from the complex at a lower ratio of BACH1^DBD^. In addition, in our experiment, NRF2^DBD^ can be almost completely displaced by BACH1^DBD^ at a high concentration of the titrant (Figure 4B).

### 3.5. Competition Assays Using Heterodimeric Constructs

**Figure 5:**
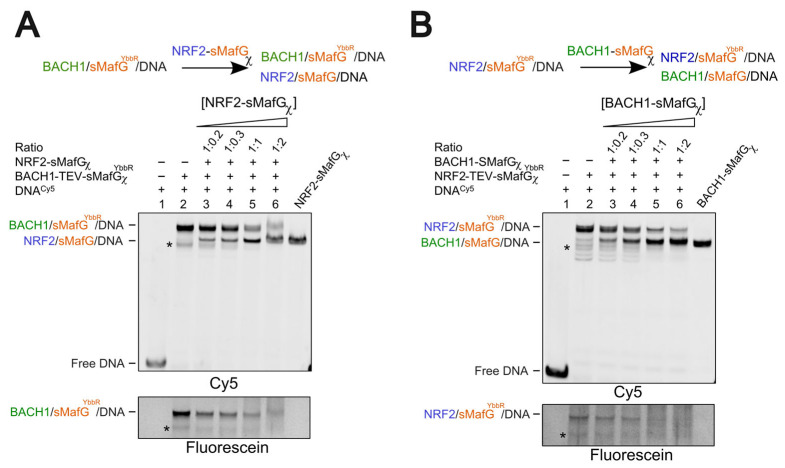
Competitive displacement by heterodimers. (**A**) Titration of NRF2/sMafG over BACH1/sMafG^YbbR^ bound on the DNA. NRF2/sMafG is able to displace BACH1/sMafG^YbbR^ from CsMBE, as demonstrated by a faster migrating complex and the disappearance of the fluorescein signal (sMafG^YbbR^). (**B**) Titration of BACH1/sMafG over NRF2/sMafG^YbbR^ bound on the DNA. BACH1/sMafG is able to displace NRF2/sMafG^YbbR^ from CsMBE, as demonstrated by a faster migrating complex and the disappearance of the fluorescein signal (sMafG^YbbR^). The ratio indicates bound complex over the titrated heterodimer (e.g., BACH1/sMafG/DNA over NRF2/sMafG in (**A**), NRF2/sMafG/DNA over BACH1/sMafG in (**B**)). As a control, DNA-bound NRF2/sMafG (**A**) and BACH1/sMafG (**B**) were used. The black asterisks represent non-specific bands.

Reports have suggested that sMafG could associate with NRF2 before binding the DNA [25]. Therefore, we tested whether heterodimeric NRF2/sMafG could effectively displace BACH1/sMafG from DNA. Increasing concentrations of NRF2-sMafGχ were added to a preformed BACH1/sMafG^YbbR^/DNA complex (Figure 5A). Upon successful competition, we expected the formation of a faster migrating complex and a concurrent loss of the fluorescein signal, indicating the co-release of BACH1^DBD^ and its tagged sMafG^YbbR^ partner from the DNA. As shown in Figure 5A, a faster migrating complex appeared at a ratio of 1:0.2 BACH1/sMafG^YbbR^: NRF2/sMafG, and a full replacement was almost complete at a ratio of 1:2. The loss of the fluorescein signal indicates that BACH1/sMafG^YbbR^ is replaced by NRF2/sMafG without exchanging sMafG^YbbR^. Furthermore, the quantity of NRF2/sMafG needed to displace BACH1/sMafG^YbbR^ is lower than when NRF2^DBD^ is used (Figure 4A). In summary, these observations suggest the successful switch between repressed and activated forms of the promoters.

We then examined the repression of the promoter by assessing whether BACH1/sMafG could displace NRF2/sMafG^YbbR^. NRF2-TEV-sMafGχ^YbbR^ was pre-incubated with DNA and increasing amounts of BACH1-sMafGχ were added (Figure 5B). Like the previous assay, a faster migrating complex appeared at a ratio of 1:0.2 NRF2/sMafG^YbbR^: BACH1/sMafG, with a nearly complete displacement at a 2-fold excess of BACH1/sMafG over NRF2/sMafG^YbbR^. Again, the decrease in the fluorescein signal indicated that BACH1/sMafG has replaced the NRF2/sMafG^YbbR^ complex altogether without swapping sMafG^YbbR^ (Figure 5B). Again, the exchange is more efficient than when BACH1^DBD^ was used (Figure 4B).

These results suggest that BACH1/sMafG and NRF2/sMafG can competitively replace each other at CsMBE-containing DNA sequences. Importantly, the displacement is more efficient when the competing transcription factors are provided as preassembled heterodimers rather than as individual bZIP domains. In the case of competing preformed heterodimers, replacement occurs without exchanging sMafG. This highlights the importance of preformed heterodimers in mediating rapid and reversible transcriptional responses to redox signals.

### 3.6. Competition Assays Using Heterodimeric Constructs Under Non-Reducing Conditions

**Figure 6:**
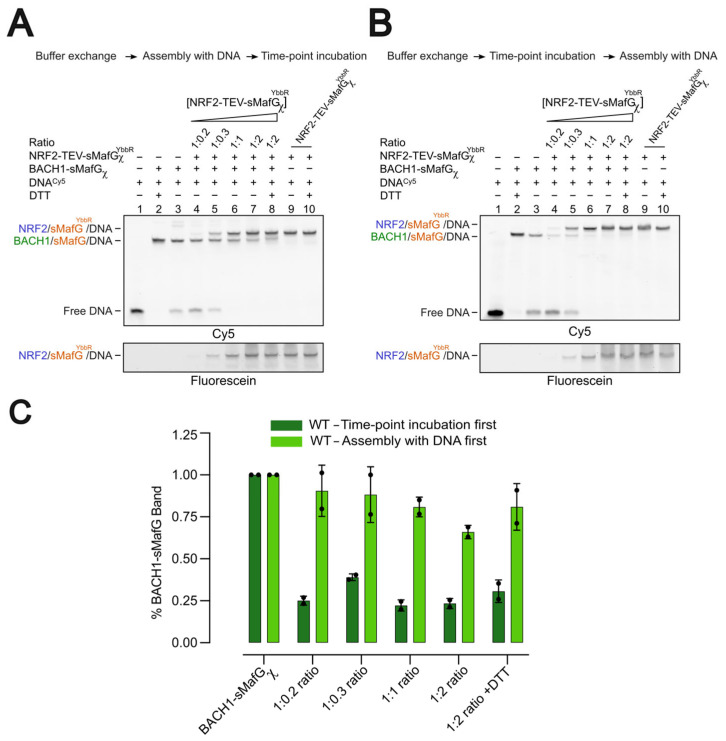
Competitive displacement by heterodimers under non-reducing conditions. Buffer-exchanged BACH1/sMafG heterodimer was either (**A**) preassembled on DNA and then incubated under non-reducing conditions for 8 h or (**B**) incubated under non-reducing conditions for 8 h and then assembled on DNA. EMSA gels of titration of NRF2/sMafG^YbbR^ over BACH1/sMafG bound on the DNA are shown. (**A**) BACH1/sMafG remained resistant to displacement by NRF2/sMafG^YbbR^. In contrast, in (**B**), NRF2/sMafG^YbbR^ efficiently displaced BACH1/sMafG complex even at low concentrations. As a control, DNA-bound NRF2/sMafG^YbbR^ and BACH1/sMafG were used in the presence and absence of DTT. (**C**) Histogram comparing the BACH1/sMafG/DNA band intensities of (**A**,**B**). Data presented are from independent duplicates.

To investigate how the redox modification of C574 influences the exchange between BACH1/sMafG and NRF2/sMafG^YbbR^, we compared the competition dynamics under two conditions: assembly first, incubation, then competition (Figure 6A) or incubation-first, assembly, then competition (Figure 6B).

Assembling BACH1/sMafG on DNA before incubation under non-reducing conditions led to a greater decrease in DNA binding (Figure 6A, lane 3) than in Figure 3B, possibly due to the longer incubation (see Materials and Methods). The addition of increasing concentrations of NRF2/sMafG^YbbR^ showed that BACH1/sMafG remained resistant to displacement (Figure 6A—lanes 4–8).

By contrast, when BACH1/sMafG was preincubated in non-reducing conditions before assembling on the DNA (Figure 6B), we did observe a large binding reduction similar to what was observed previously (Figure 3A). Adding the NRF2/sMafG^YbbR^ efficiently displaced the BACH1/sMafG complex at lower concentrations compared to the assembly first experiment (Figure 6A). Quantification of the BACH1/sMafG/DNA complex band intensities confirmed this trend (Figure 6C). When the band intensities are scaled to the quantity of the BACH1/sMafG bound before adding NRF2/sMafG^ybbR^ (Figure 6A,B—lane 3), a large drop in the BACH1/sMafG/DNA band intensity is observed in the incubation-first experiment (Figure 6B). This shows that the complex that was assembled on the DNA first is not as easily displaced by NRF2/sMafG^YbbR^ compared to the complex that was incubated in non-reducing conditions first.

These observations suggest that the modifications occurring under non-reducing conditions when BACH1/sMafG is off the DNA either do not take place once the complex is bound or that C574 is specifically protected from redox modifications when engaged on the DNA.

## 4. Discussion

In this study, we employed a chimeric tethering strategy combined with covalent fluorescent labelling to dissect the DNA-binding dynamics and competitive interplay between repressive BACH1/sMafG and activating NRF2/sMafG heterodimers. By covalently linking the DNA-binding domains of BACH1^DBD^ and NRF2^DBD^ to sMafG^DBD^ using a protease-cleavable linker, we enforced heterodimer formation and overcame the kinetic limitations associated with partner exchange in vitro. This experimental design allowed us to quantitatively assess DNA-binding affinities, redox sensitivity, and the displacement behaviour of various bZIP dimers at CsMBEs. Importantly, the sequence chosen as well as the flanking regions are found to be bound by both BACH1 and NRF2 in ChIP-seq experiments [21,22]. Therefore, our results are likely to be generalisable to other all CsMBE sequences.

Our results demonstrate that both tethered NRF2/sMafG and BACH1/sMafG heterodimers bind CsMBE sequences with a high affinity, surpassing those of their respective bZIP homodimers. This finding validates the utility of our approach for dissecting stable heterodimer/DNA interactions. A similar tethering method has previously been applied to study sMafG/NRF2 in cells [30]; here, we extend their applicability to BACH1/sMafG. The inclusion of fluorescently labelled sMafG^YbbR^ enabled the tracking of partner replacements in EMSA, offering a precise readout for the dynamics of heterodimer exchange. This modular and broadly applicable system could be adapted to other bZIP pairings, providing a powerful means to investigate DNA-binding competition and exchange under defined and tuneable biochemical conditions.

Our data show that, like sMafG^DBD^, BACH1^DBD^ can form homodimers in a solution in the absence of DNA. This dimerisation is likely an artefact of recombinant expression: the leucine zipper presents an apolar surface that promotes dimer formation, with surrounding residues further contributing to the interaction [28]. In contrast, NRF2^DBD^ showed no evidence of dimerisation under the same conditions, likely due to its predominantly disordered nature [34]. Consistently, the NMR structure of a construct comprising only the CNC and basic regions (Figure S1A) shows that the CNC domain is folded, while the basic region is disordered (PDB ID 2LZ1).

In the cellular context, there is compelling evidence that NRF2 associates with sMafG in the nucleoplasm, even in absence of DNA binding. A study by Li et al. showed that the leucine zipper of NRF2 can associate with sMafG in cells, enhancing the nuclear retention of NRF2 by masking its NESzip motif [35]. This suggests that NRF2/sMafG heterodimerisation plays a role in the subcellular localisation independent of DNA binding. While earlier in vitro biochemical data demonstrated that BACH1 binds sMafG in the presence of DNA [7], cellular interaction off the DNA should be assessed in the future. Consistent with the idea that NRF2 (and possibly BACH1) associate with sMafG off the DNA in cells, our competition assays using preassembled heterodimers demonstrate that BACH1/sMafG can displace NRF2/sMafG from the DNA with similar efficiency as NRF2/sMafG displaces BACH1/sMafG (Figure 5A,B). In both cases, comparable amounts of titrants are sufficient to achieve displacement. This supports the notion that relative protein abundance is one of the key determinants of transcriptional regulation at CsMBE promoters. This is particularly relevant given that both NRF2 and BACH1 are regulated at the protein level by E3 ligases [9,10,36].

We observed that the displacements of DBDs from the DNA are more efficient when they are assembled as heterodimers. This can be explained by the structural and thermodynamic features inherent to bZIP architecture. Indeed, when BACH1^DBD^ competes with NRF2 in a NRF2/sMafG complex, the process likely requires dissociation of the homodimer, which imposes an energetic barrier. Once displaced, NRF2^DBD^ might not be as stable than when engaged with sMafG. Likewise, if indeed NRF2^DBD^ is disordered in solution, folding of the basic region—and potentially the leucine zipper—would carry an additional energetic cost required to displace BACH1^DBD^ from a BACH1/sMafG complex. Similarly, when displaced, BACH1^DBD^ will have the hydrophobic leucine zipper exposed, which is likely to be less stable than when engaged in a dimer. These considerations support a model in which preassembled heterodimers are thermodynamically and kinetically favoured for the efficient bZIP exchange at CsMBE elements, at least in in vitro conditions.

A central finding of this study is that BACH1’s DNA-binding capacity is impaired in a C574-dependent manner. Free BACH1/sMafG heterodimer incubated under non-reducing conditions showed diminished DNA-binding competence, whereas the preformed BACH1/sMafG/DNA complex remained stable (Figure 3B,C). These observations suggest that redox modifications at C574 either occur only when BACH1/sMafG is off the DNA or that C574 is protected once engaged on the DNA. Structural modelling places C574 adjacent to the DNA phosphate backbone, where oxidative modifications could sterically or electrostatically interfere with DNA contacts. Our competition assays further support this model: when oxidation preceded DNA binding, the displacement of BACH1/sMafG by NRF2/sMafG was markedly accelerated, but once assembled on the DNA, the complex resisted oxidation (Figure 6C).

These findings are consistent with earlier work by Ishikawa et al. [12], who showed that electrophiles such as diamide and HNE impair BACH1–DNA binding in cells, implicating C574 as a key regulatory site. Direct comparison, however, is challenging because of key differences in the experimental design: our assays use a reconstituted system containing only the DNA-binding domains, whereas Ishikawa’s observations were made in cells with the full-length protein. It therefore remains possible that electrophiles promote BACH1 release indirectly—for example, through heme release—and that C574 is subsequently modified in the unbound protein, thereby preventing re-binding. Alternatively, the redox modifications observed in our system may occur only when BACH1 is off DNA and not when it is engaged with sMafG on the DNA. Together, the cellular findings of Ishikawa and our biochemical data support a unified model in which the redox modifications of C574 attenuate the ability of BACH1 to (re)engage CsMBE sites.

## 5. Conclusions

Our work provides a mechanistic framework for understanding CNC-bZIP transcriptional switching at CsMBEs. By combining a chimeric system and a redox-sensitive mutation strategy, we have identified key principles governing bZIP partner exchange on DNA. These include the stabilising role of preformed heterodimers, the thermodynamic constraints on monomeric intermediates during displacement, and the role of C574 oxidation in modulating the DNA binding of BACH1. More broadly, our findings underscore how subtle post-translational modifications—including thiol oxidation—layer regulatory complexity atop DNA-binding networks, enabling a nuanced and reversible transcriptional control in response to the redox status.

## Data Availability

The original contributions presented in this study are included in the article/Supplementary Materials. Further inquiries can be directed to the corresponding author.

## Supplementary Materials

The following supporting information can be downloaded at: https://www.mdpi.com/article/10.3390/antiox14101203/s1, Figure S1: Sequence alignment of the DNA-binding domains; Figure S2: BACH1^DBD^ and sMafG^DBD^ oligomerisation state; Figure S3: NRF2^DBD^ and sMafG^DBD^ interaction with CsMBE; and Figure S4: Close-up views of C574 of BACH1 (AlphaFold model), C361 of Brf2 (PDB ID 4ROC [33]) and C59 of NF-kB (PDB ID 1NFK [32]).

## Abbreviations

The following abbreviations are used in this manuscript:

DBD	DNA Binding Domain
EMSA	Electrophoretic Mobility Shift Assay
