# Supplementary material for: Dissecting the Interplay Between NRF2 and BACH1 at CsMBEs
Source: Antioxidants (Basel). 2025 Oct 3;14(10):1203. doi: 10.3390/antiox14101203 (PMC12561426; doi:10.3390/antiox14101203)
Supplement: Supplementary file 1 [file antioxidants-14-01203-s001.zip › antioxidants-3863540-supplementary.pdf]

Supplementary Figures

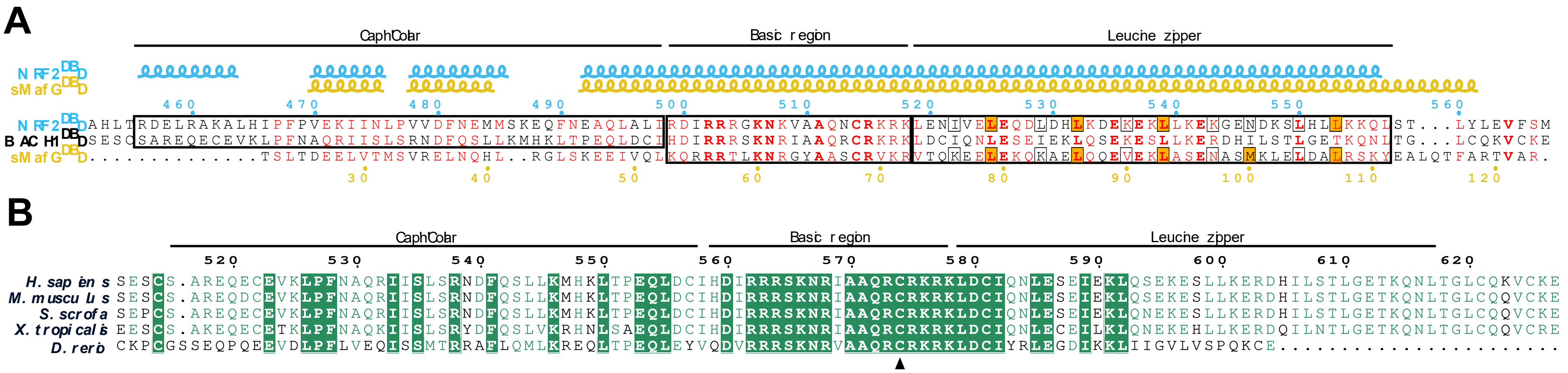

Figure S1. Sequence alignment of the DNA-binding domains. (A) Alignment of the Nrf2DBD, Bach1DBD, and sMafGDBD sequences. Identical residues are shown in bold red, while similar residues appear in red. The secondary structures of Nrf2 and sMafG (from [18]) are indicated above the alignment. Major domains are outlined with large boxes. Residues involved in dimerization are enclosed in black boxes, and the leucine zipper region is additionally highlighted with a yellow background. (B) Alignment of Bach1 from multiple species. Similar residues are highlighted in green, while identical residues are shown in bold white text on a green background.

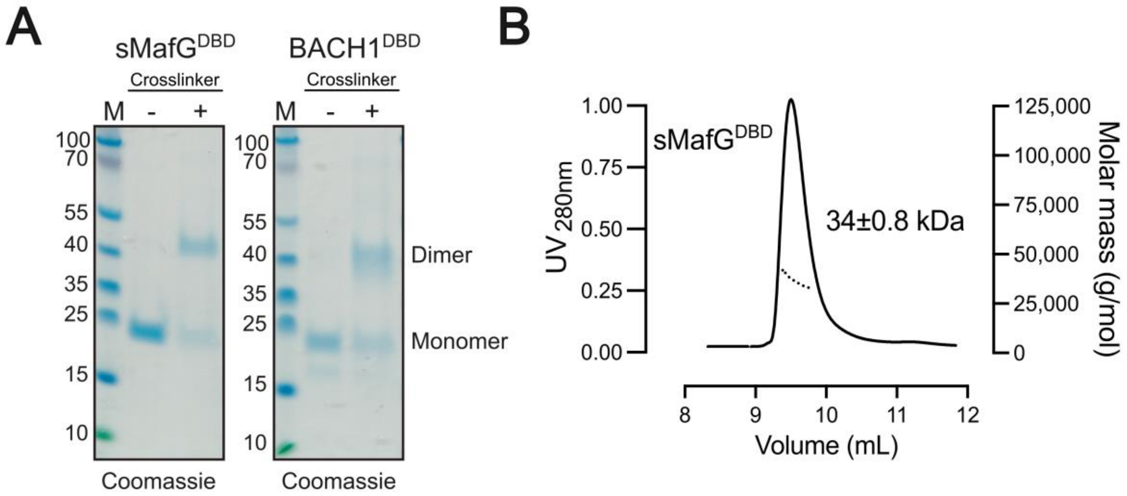

Figure S2. Bach1^{DBD} and sMafG^{DBD} oligomerisation state. **(A)** Crosslinking using hanging-drop vapor diffusion method used to study the oligomeric states of Bach1^{DBD} and sMafG^{DBD}. Glutaraldehyde crosslinked samples show increased apparent molecular weight on SDS-PAGE gel, indicating that recombinant proteins Bach1^{DBD} and sMafG^{DBD} form homodimers. **(B)** SEC-MALS chromatogram of sMafG^{DBD} (34±0.8 kDa) shows the homodimeric state of the recombinant protein.

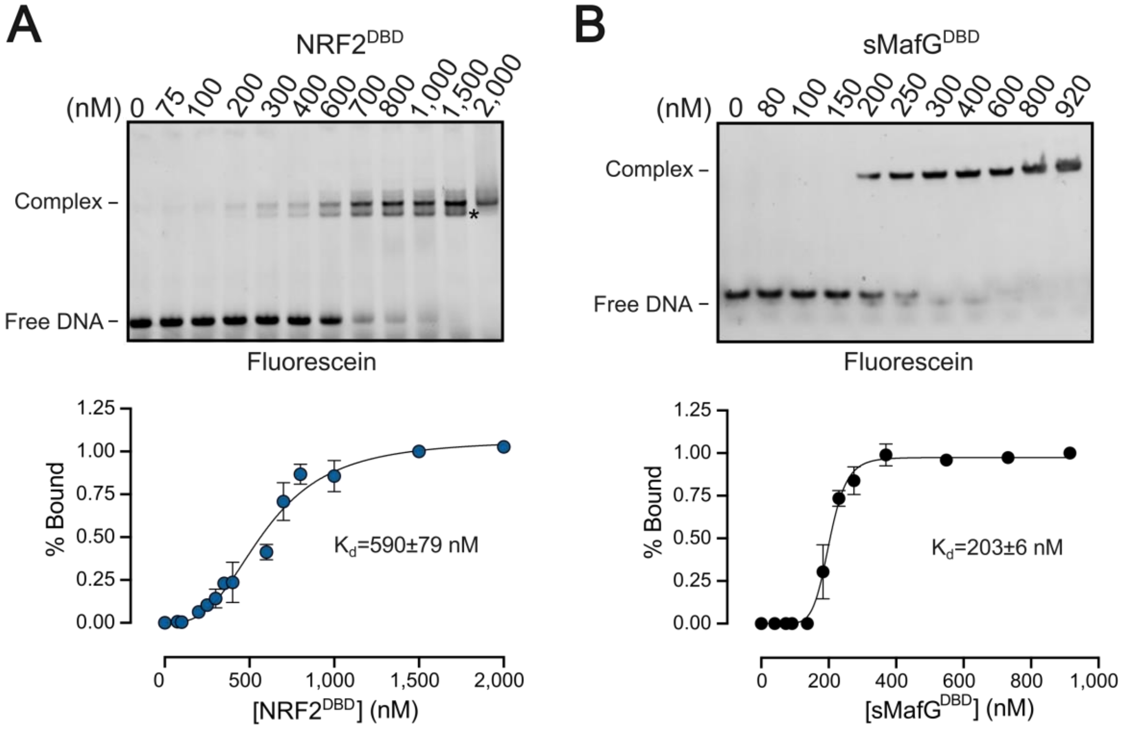

Figure S3. Nrf2^{DBD} and sMafG^{DBD} interaction with CsMBE. Fluorescence-based EMSA gels of **(A)** Nrf2^{DBD} and **(B)** sMafG^{DBD} using a fluorescein labelled DNA are shown. The complex and the free DNA bands are indicated. The concentration values represent the concentration of the homodimeric form of the proteins. The band intensity analysis is shown. The affinity of Nrf2^{DBD} and sMafG^{DBD} binding to CsMBE is estimated to be around 590±79 nM and 203±6 nM, respectively. The black asterisk represents non-specific bands. Data presented are from three independent replicates.

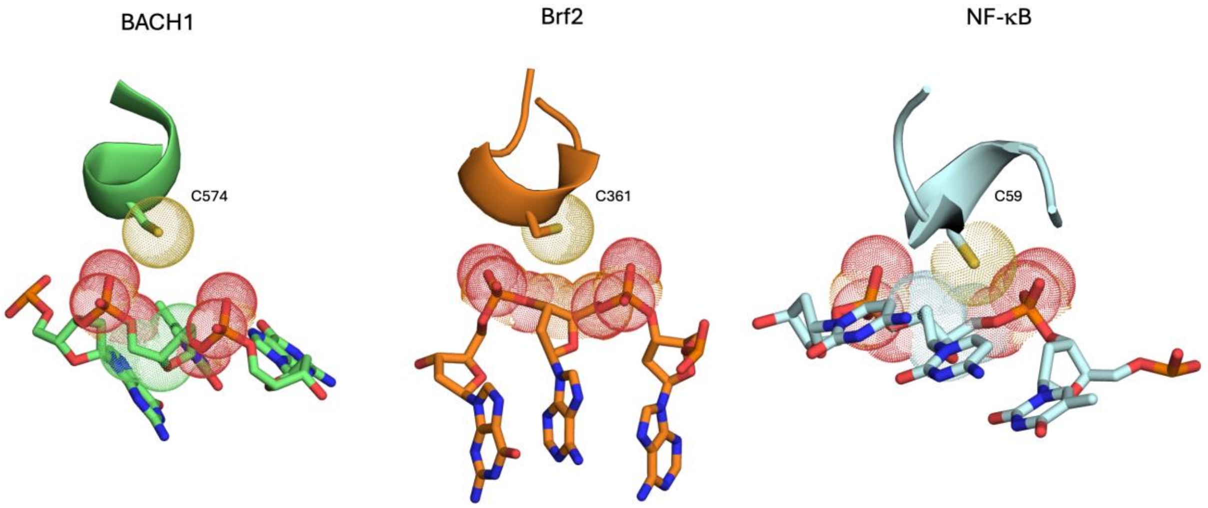

Figure S4. Close-up views of C574 of BACH1 (AlphaFold model), C361 of Brf2 (PDB ID 4ROC [31]) and C59 of NF-κB (PDB ID 1NFK [30]). The thiol function of the cysteine is located near the DNA, forming Van der Waals contact. The dot radius represents the Van der Waals radius of each atom.
